# Perceptions of surgery in Nicaragua: A cross-sectional survey study within the surgery for the people project

**DOI:** 10.1371/journal.pgph.0003835

**Published:** 2024-10-17

**Authors:** John Dutton, Neil Parikh, Maria Cabrera, Carolina Robleto, Mikyla Lambert, Emily Jones, Sonia Treminio, Dory Barkhordarzadeh, Allyn Auslander, Ruben Ayala

**Affiliations:** 1 Operation Smile Inc., Virginia Beach, Virginia, United States of America; 2 Rutgers University, New Brunswick, New Jersey, United States of America; 3 University of Southern California, Los Angeles, California, United States of America; 4 Operation Smile Nicaragua, Managua, Nicaragua; University of Global Health Equity, RWANDA

## Abstract

Barriers to medical care include lack of proper infrastructure and equipment; however, cultural barriers to care and poorly perceived quality of care, especially surgical care, can also negatively impact a patient’s utilization of healthcare services. This study used patient-survey data from three unique municipalities in Nicaragua to examine pre-hospital barriers to care, including previous experience with healthcare, and how those experiences impact patient perceptions of surgery and care-seeking behavior. Surveys were administered in Siuna, Rosita, and Bonanza, Nicaragua between July 2019 and September 2020. Survey participants were aged 18-years or older that live in communities served by the Ministry of Health. The surveys were open response and multiple-choice format. Surveys included questions about structural/cultural/financial barriers to care, communication barriers, and knowledge of healthcare services. Data was managed using REDCap tools and analysis was completed using R. Individuals that previously visited a health post were significantly more likely to have a positive perception of surgery compared to those who had not (OR = 1.4) (p = 0.019). This finding remained significant after adjustment for education, age, and municipality. However, previous hospital visits did not have a significant impact on perception of surgery. Individuals with higher transportation costs reported a negative perception of surgery (40.4%), as well as those who used private transportation (29.1%) (p<0.001). Participants that reported travel obstacles were 2.64 times as likely to have a positive perception of surgery (p<0.001), even when adjusted for all demographics except site. These findings suggest that individuals who previously interacted with only lower-level healthcare environments were significantly more likely to have a positive perception of surgery. Counterintuitive findings show that access to public transport, transportation costs >2USD, and cell-phone usage increased negative perception of surgery. This study demonstrates the complexity of variables that impact perceptions of healthcare services while highlighting areas of focus for future targeted investments.

## Introduction

In 2015, an estimated 5 billion individuals worldwide lacked access to safe and affordable anesthesia or essential surgical care due to structural, financial, and cultural barriers [1,2]. Despite 170 million operations being performed annually, it was estimated that 2.3 million surgical cases were cancelled per week worldwide during the peak of the COVID pandemic, with 8.4% of weekly cancelled cases occurring in low- and middle-income countries (LMICs) [3]. The inequity in essential healthcare services, coupled with decreased access due to a global pandemic, is a burden that is disproportionately shouldered by residents within low resource environments. Adequate access to safe surgical care is not only determined by funding, infrastructure and resource management but is also believed to be influenced by the person’s willingness to seek care, as outlined by the Lancet Global Health 3 delay framework [4]. Prior research suggests that a patient’s perceived quality of care directly influences their utilization of healthcare services [5]. However, the specific elements that contribute most to this perceived quality, particularly of surgery, remain poorly elucidated.

A country’s surgical capacity is determined by a variety of factors such as funding, equipment, and personnel. The surplus or paucity of these components affect the availability and accessibility of surgical services; however, supply-side constraints are only a piece of the complete picture. It is equally important to understand how a patient has previously accessed or witnessed others accessing care. These experiences can alter a patient’s perception and affect changes in demand and care seeking behavior, even when care is available. Existing data supports the theory that patients may delay seeking healthcare due to cultural beliefs, a lack of prior exposure to health systems, and a low awareness or confidence in existing health services [2]. Additionally, perceived quality may be influenced by patient demographic and geographic factors, health literacy, and public discourse [6,7].

While many existing studies contribute to our understanding of healthcare underutilization and provide direction for improving access to care in LMICs, research focused on surgical service utilization is critical to fully address this multi-dimensional relationship. This study examines the perception of surgery in various patient groups across different healthcare environments to understand how perceived quality may impact timely and appropriate care-seeking behavior. Specifically, this study uses patient-survey data to examine the association between a patient’s pre-hospital barriers, including their prior experience with different healthcare environments, and the impact these exposures have on a participant’s perception of surgical care in three municipalities in Nicaragua. This study has wide-reaching implications to help provide insight and guide demand-side investments aimed at encouraging care seeking behavior and increasing surgical service utilization in LMICs.

### Nicaragua overview

Nicaragua is the poorest country in Central America. While the country experienced a drastic economic contraction, from 2018 to 2020 due to socio-political challenges, there has been significant recovery with GDP rebounding by 11.6% in 2021 and another 10.7% in 2022 [8]. Poverty has decreased to 13.3% in 2022 from 14.2% in 2021; with a large spike in emigration likely contributing this this improvement [8]. Despite this improved outlook within the country, Nicaragua still only has 0.9 hospital beds per 1000 people, 15.8 surgeons, anesthesiologists and obstetricians (SAOs) per 100,000 population and performs 4,860 surgical procedures per 100,000 population [9,10]. These numbers sit below the minimum targets of 20 SAOs and 5,000 surgical procedures as set out by Lancet Commission on Global Surgery [4], however these estimates do not portray a realistic picture of Nicaragua as a whole, as the shortage disproportionately affects the Eastern half of the country. The Las Minas region, a mining triangle in Nicaragua’s northern autonomous region with 169,000 inhabitants, has an SAO density of approximately 7/100,000 population and a patient annual case volume of approximately 1,000 procedures per 100,000 population.

### Study setting

The study is composed of groups from different rural communities and urban sectors from Siuna, Rosita and Bonanza. These municipalities are located in the mining triangle “Las Minas”, in the Caribbean Coast of Nicaragua’s North Autonomous Region. There are three hospitals within the Las Minas region, all providing basic and emergency level care within the municipalities of Siuna, Rosita and Bonanza. The closest hospitals that provide more specialized care are a 10–15 hour drive away, depending on road conditions. Less than 50% of individuals within the Las Minas region can reach their closest hospital within 2 hours.

The municipality of Siuna is composed of approximately 105,000 residents of which 77.5% are rural. Most of the inhabitants rely on 17 health posts for basic medical necessities. Similarly, Rosita consists of approximately 36,800 individuals of which 58.2% are rural. The Rosario Pravia Medina Hospital provides primary health care to one third of Rosita’s population with the remaining 24,000 inhabitants relying on 10 health posts, three private clinics, and one public clinic. Finally, Bonanza is home to approximately 29,800 individuals of which 52.6% are rural. There are 10 health posts within Bonanza that provide care and support to most of the rural population in this municipality. There are a total of 855 health posts across Nicaragua, with twice as many health posts in the pacific and central region compared to the Caribbean region. Health posts are under the jurisdiction of health centers, but both provide care at the municipal level. Health posts are staffed by 1–2 nurses, and sometimes a physician on a temporary basis. Health posts provide basic primary healthcare and take on both health promotion and disease prevention for their surrounding communities [11].

## Methods

The described study was part of a larger health system strengthening project, pioneered by Operation Smile in 2017 and funded by the UBS Optimus Foundation in collaboration with the Ministry of Health in Nicaragua. The Surgery for the People Project leveraged Operation Smile’s 38 years of experience partnering with local teams in LMICs to deliver surgical care to vulnerable populations.

### Study design and period

The study was cross-sectional in design following the STROBE checklist available in the supplemental materials. Data was gathered in Siuna from July 2—July 4, 2019, in Bonanza from July 5—July 6, 2019, and finally in Rosita from September 9—September 11, 2020.

### Sample size determination

Sample size was determined across each municipality by identifying community and sector populations served by the Ministry of Health "SILAIS Las MINAS”. This information was cross referenced with the Institute of Development (INIDE) report from 2005 and 2016, including both municipality population and the population growth rate per year. Sample sizes for survey participants were calculated with a 5% margin of error based on the estimated adult populations of each sector where the community was located within each municipality.

In total, nine rural communities and three urban neighborhoods (3 rural and 1 urban per municipality) were identified for recruitment of participants and survey participation. Local healthcare workers who aid the surrounding rural communities were consulted to assure broad and equitable inclusion of communities and neighborhoods with similar geographical distribution (i.e. distance from each hospital) across municipalities.

### Measurement

Surveys included open response and multiple-choice questions to evaluate pre-hospital structural, cultural, financial, and transportation barriers and the participants’ perceptions of surgery. The survey had 35 questions organized into 5 sections: general information (15 questions), structural/cultural/financial barriers to care (10 questions), communication barriers (5 questions) and knowledge and perceptions of available healthcare services, specifically surgery (5 questions). Surveys were created in English by local doctors, nurses and community health workers that were employed by Operation Smile, Nicaragua. The finalized survey was then reviewed with the monitoring and evaluation team of Operation Smile International prior to participant recruitment. Surveys were subsequently translated to Spanish by the local research team. Additional translation was needed to accommodate local dialects. Translation back to English was done to confirm consistency across all dialects. Upon completion of surveys within each of the sampled communities, survey data was managed using REDCap electronic data capture tools hosted by Operation Smile. REDCap (Research Electronic Data Capture) is a secure, web-based software platform designed to support data capture for research studies.

### Sampling technique and data collection procedure

All team members administering surveys were extensively trained in managing data collection and interview techniques to maintain consistency across sites. Within each municipality, supervisors of the data collection teams were assigned to provide guidance on where to administer surveys and monitor the total number of surveys conducted. Surveys were administered verbally to participants at their home and completed on paper by the data collection teams. Survey collection was performed in this manner to avoid obstacles with literacy and increase overall respondent participation upon obtaining verbal consent.

Upon arriving in each community, the lot quality assurance sampling (LQAs) technique was applied to assure a systematic sampling approach to count homes and verify that the survey distribution was appropriate and adequately covered the entire community. This approach ensured that captured surveys provided a representative picture of the community, minimized biases, and increased the reliability of the collected data.

### Survey variables

*Survey questions with open response were grouped for ease of analysis*. *Job types were grouped as informal if the respondents job was not regulated by the local or national government and/or were not associated with benefits other than an hourly wage*. Participants reported having travel obstacles in a dichotomous manner, yes or no, but survey participants were prompted by data collectors that obstacles included obstructions such as streams, rivers, or a lack of physical roads if respondents asked for clarification on the definition of “obstacles”. Transportation cost to a hospital or health post was collected with an open response. These responses were then categorized as < or > 60 Cordobas (approximately $2USD as of 2020) and the typical cost of a bus fare in Nicaragua, but with a range between 30–120 Cordobas (approximately $1-$4USD) as verified by the local research team. The perception of surgery was dichotomized into positive compared with negative and neutral (combined). We allowed participants a “neutral” option to not force respondents to choose an answer, thus potentially causing incorrect data, and to allow them to answer our main question of interest and potentially prevent a non-response, thus causing missing data.

### Eligibility criteria

Once a home was selected, sampling by convenience was used to ask an individual within that home about their willingness to participate. Individuals would then be surveyed only if they met the eligibility criteria. The inclusion criteria consisted of individuals aged 18 years or older residing in the selected urban neighborhoods and rural communities of Siuna, Rosita, and Bonanza. Individuals not residing in these specified areas and those working for or having a relative working for the public health system were excluded from the survey.

### Statistical analysis

Descriptive statistics, including proportions for categorical variables and means for continuous variables, were constructed for both personal and healthcare related characteristics of participants by site and perception of surgery. Tests of statistical significance included t-tests for continuous and χ2 tests for categorical variables. P-values were considered significant when p < 0.05.

Logistic regression was used to calculate odds ratios and 95% confidence intervals for the relationship between perception of surgery and key barriers to care. Unadjusted and adjusted odds ratios are reported based on known confounders and covariates of interest. All odds ratios were considered significant when p-values were less than 0.05. All analyses were completed using R (R Foundation for Statistical Computing, Vienna, Austria).

### Ethical consideration

Ethical approval for the study was obtained from the Institutional Review Board at the University of Southern California (CHLA-20-00027). Verbal consent was obtained from all surveyed participants prior to the interview, witnessed by supervisors, and documented in writing on the survey which was an approved method by the IRB. There was no risk or harm to participants, and they were made aware that participation was optional. Surveys were anonymous and no personal identifiers were used. Data was accessed for research purposes on August 31, 2022.

## Results

The sample consisted of 1132 individuals overall that were available for analysis. In total 32.8% (n = 372) of the sample was from Bonanza, 33.4% from Rosita (n = 378), and 33.7% from Siuna (n = 382).

The demographic characteristics by site are seen in Table 1. The age structure, rural to urban ratio, education, and job type all differed significantly by site (p < 0.001). Bonanza was the youngest population followed by Siuna (32.2%) and Rosita (27.0%). Although the majority of all participants lived in rural areas across all sites, Siuna had the largest rural population (n = 334, 87.4%) followed by Rosita (n = 240, 63.5%) and Bonanza (n = 226, 60.8%). In Siuna, 86.1% of the participants had a primary school education or less (n = 329), followed by Bonanza (n = 268, 72.0%) and Rosita (n = 236, 62.4%). Most participants were employed in the informal sector for Bonanza and Siuna (n = 218, 58.6%; and n = 193, 51.1% respectively) whereas unemployment was the most common response in Siuna (n = 178, 46.6%). Most individuals in all sites were married (n = 834, 73.7%), female (n = 781, 69%), and the most common household size was 4 to 6 (n = 561, 49.6%) with no difference by site.

**Table 1:** Demographics across all sites.

	Bonanza	Rosita	Siuna	Total	P-Value
Number of Surveys (n)	372	378	382	1132	
**Age**					<0.001*
** 18–34 years old**	213 (57.3%)	141 (37.3%)	168 (44%)	522 (46.1%)	
** 35–59 years old**	137 (36.8%)	197 (52.1%)	169 (44.2%)	503 (44.4%)	
** >60 years old**	22 (5.9%)	40 (10.6%)	45 (11.8%)	107 (9.5%)	
**Gender**					**0.19**
** Male**	95 (25.5%)	130 (34.4%)	126 (33%)	351 (31%)	
** Female**	277 (74.5%)	248 (65.6%)	256 (67%)	781 (69%)	
**Rural/Urban**					<0.001*
** Rural**	226 (60.8%)	240 (63.5%)	334 (87.4%)	800 (70.7%)	
** Urban**	146 (39.2%)	138 (36.5%)	48 (12.6%)	332 (29.3%)	
**Education Level**					<0.001*
** Primary or less**	268 (72%)	236 (62.4%)	329 (86.1%)	833 (73.6%)	
** Secondary or more**	104 (28%)	142 (37.6%)	53 (13.9%)	299 (26.4%)	
**Job Type**					<0.001*
** Formal**	22 (5.9%)	43 (11.4%)	89 (23.3%)	154 (13.6%)	
** Informal**	218 (58.6%)	193 (51.1%)	115 (30.1%)	526 (46.5%)	
** Unemployed**	132 (35.5%)	142 (37.6%)	178 (46.6%)	452 (39.9%)	
**Marriage Status**					**0.078**
** Divorced/Widowed**	13 (3.5%)	23 (6.1%)	18 (4.7%)	54 (4.8%)	
** Married/Domestic Partnership**	293 (78.8%)	268 (70.9%)	273 (71.5%)	834 (73.7%)	
** Single**	66 (17.7%)	87 (23%)	91 (23.8%)	244 (21.6%)	
**Household Size**					**0.729**
** 0 to 3**	142 (38.2%)	127 (33.6%)	141 (36.9%)	410 (36.2%)	
** 4 to 6**	179 (48.1%)	197 (52.1%)	185 (48.4%)	561 (49.6%)	
** 7 or more**	51 (13.7%)	54 (14.3%)	56 (14.7%)	161 (14.2%)	

Table 2 highlights health related characteristics of the population. All variables were significantly different by site (p < 0.01). 61.0% of Bonanza’s population reported having never visited a health post (n = 227) compared to 43.9% (n = 166) of Rosita and 18.1% (n = 69) of Siuna. Overall, 40.8% (n = 462) of the population have never visited a health post prior and 81% (n = 917) visited a hospital. Of those who supplied cost responses, most participants in Rosita and Siuna (n = 168, 44.4%; and n = 200, 52.4%, respectively) paid more than 60 Cordoba to reach the health post while only 16.4% (n = 61) of Bonanza paid over 60 Cordoba. 47.0% (n = 175) of Bonanza respondents, 46.3% (n = 175) of Rosita respondents, and 51.6% (n = 197) of Siuna respondents used public transportation. Private transportation was more common in Rosita (36%) compared to Bonanza (18.5%) and Siuna (6.8%). Participants in both Bonanza and Rosita more often had less than one hour of travel to the closest hospital (n = 277, 74.5%; and n = 211, 55.8%, respectively), whereas 52.1% (n = 199) of participants in Siuna had over an hour of travel. The majority of participants in Bonanza (n = 292, 78.5%) reported facing travel obstacles compared to 11.4% (n = 43) in Rosita and 17.0% (n = 65) in Siuna. When assessing participants’ perception of surgery, 75.4% (n = 288) of Siuna’s participants stated a positive perception followed by 70.7% (n = 263) of Bonanza and 50.8% (n = 192) of Rosita. Most participants had a cellphone with 75.4% (n = 285) of participants in Rosita followed by 74.9% (n = 286) in Siuna and 62.1% (n = 231) in Bonanza.

**Table 2:** Health-related variables by site.

	Bonanza	Rosita	Siuna	Total	P-Value
Number of Surveys (n)	372	378	382	1132	
**Health Post Visit**					<0.001*
** Yes**	145 (39%)	211 (55.8%)	313 (81.9%)	669 (59.1%)	
** No**	227 (61%)	166 (43.9%)	69 (18.1%)	462 (40.8%)	
** Missing**	0 (0%)	1 (0.3%)	0 (0%)	1 (0.1%)	
**Hospital Visit**					<0.001*
** Yes**	338 (90.9%)	312 (82.5%)	267 (69.9%)	917 (81%)	
** No**	34 (9.1%)	66 (17.5%)	115 (30.1%)	215 (19%)	
**Transportation Cost**					<0.001*
** <60 cordoba**	277 (74.5%)	30 (7.9%)	111 (29.1%)	418 (36.9%)	
** 60 Cordoba or more**	61 (16.4%)	168 (44.4%)	200 (52.4%)	429 (37.9%)	
** Missing**	34 (9.1%)	180 (47.6%)	71 (18.6%)	285 (25.2%)	
**Transportation Type**					<0.001*
** Private**	69 (18.5%)	136 (36%)	26 (6.8%)	231 (20.4%)	
** Public**	175 (47%)	175 (46.3%)	197 (51.6%)	547 (48.3%)	
** Other**	94 (25.3%)	1 (0.3%)	44 (11.5%)	139 (12.3%)	
** Missing**	34 (9.1%)	66 (17.5%)	115 (30.1%)	215 (19%)	
**Transportation Time**					<0.001*
** Less than 1 hr**	277 (74.5%)	211 (55.8%)	68 (17.8%)	556 (49.1%)	
** 1 hr or more**	61 (16.4%)	101 (26.7%)	199 (52.1%)	361 (31.9%)	
** Missing**	34 (9.1%)	66 (17.5%)	115 (30.1%)	215 (19%)	
**Public Transportation Available**					<0.001*
** Yes**	204 (54.8%)	371 (98.1%)	197 (51.6%)	772 (68.2%)	
** No**	168 (45.2%)	6 (1.6%)	185 (48.4%)	359 (31.7%)	
** Missing**	0 (0%)	1 (0.3%)	0 (0%)	1 (0.1%)	
**Travel Obstacles Exist**					<0.001*
** Yes**	292 (78.5%)	43 (11.4%)	65 (17%)	400 (35.3%)	
** No**	46 (12.4%)	333 (88.1%)	204 (53.4%)	583 (51.5%)	
** Missing**	34 (9.1%)	2 (0.5%)	113 (29.6%)	149 (13.2%)	
**Opinion on Surgery**					<0.001*
** Positive**	263 (70.7%)	192 (50.8%)	288 (75.4%)	743 (65.6%)	
** Negative/Neutral**	27 (7.3%)	171 (45.2%)	94 (24.6%)	292 (25.8%)	
** Missing**	82 (22%)	15 (4%)	0 (0%)	97 (8.6%)	
**Cellphone Use**					<0.001*
** Yes**	231 (62.1%)	285 (75.4%)	286 (74.9%)	802 (70.8%)	
** No**	141 (37.9%)	93 (24.6%)	96 (25.1%)	330 (29.2%)	

In addition to analyzing demographic and health related variables by site, we assessed differences across these characteristics by perception of surgery (positive vs. negative/neutral) (Tables 3 and 4). Although the age distribution varied by site, there was no difference in perception of surgery by age (p = 0.62). A higher proportion of individuals with a negative perception of surgery lived in urban areas compared to those with positive perception (p < 0.001). Individuals who were married and had a primary education were proportionally more likely to have a positive perception of surgery (p < 0.01). There was no statistically significant difference in perception of surgery by job type, gender, or household size (p > 0.05). Individuals who had been to a health post were more likely to have a positive perception of surgery compared to those who had not (p = 0.02), however no significant difference was seen among those who reported having visited a hospital (p = 0.076). A higher proportion of individuals with transportation costs greater than 60 Cordobas (~$2 USD) reported a negative perception of surgery as well as those who reported using private transportation (p < 0.01 for both). A higher proportion of individuals with a negative perception reported having access to public transportation (p < 0.01) and a cell phone (p = 0.04). Interestingly, a higher proportion of individuals with a positive perception of surgery reported travel obstacles than those with a negative opinion (p < 0.01). There was no statistically significant difference in opinion of surgery by transportation time (p > 0.05).

**Table 3:** Perceptions of surgery, stratified by demographics.

	Positive	Neutral/Negative	Total	P-Value
Number of Respondents (n)	743	292	1132	
**Age**				**0.62**
** 18–34 years old**	341 (45.9%)	131 (44.9%)	522 (46.1%)	
** 35–59 years old**	333 (44.8%)	128 (43.8%)	503 (44.4%)	
** >60 years old**	69 (9.3%)	33 (11.3%)	107 (9.5%)	
**Gender**				**0.977**
** Male**	234 (31.5%)	91 (31.2%)	351 (31%)	
** Female**	509 (68.5%)	201 (68.8%)	781 (69%)	
**Rural/Urban**				<0.001*
** Rural**	556 (74.8%)	186 (63.7%)	800 (70.7%)	
** Urban**	187 (25.2%)	106 (36.3%)	332 (29.3%)	
**Education Level**				**0.038**
** Primary or less**	560 (75.4%)	201 (68.8%)	833 (73.6%)	
** Secondary or more**	183 (24.6%)	91 (31.2%)	299 (26.4%)	
**Job Type**				**0.410**
** Formal**	107 (14.4%)	41 (14%)	154 (13.6%)	
** Informal**	326 (43.9%)	141 (48.3%)	536 (46.5%)	
** Unemployed**	310 (41.7%)	110 (37.7%)	452 (39.9%)	
**Marriage Status**				<0.001*
** Divorced/Widowed**	29 (3.9%)	21 (7.2%)	54 (4.8%)	
** Married/Domestic Partnership**	567 (76.3%)	190 (65.1%)	834 (73.7%)	
** Single**	147 (19.8%)	81 (27.7%)	244 (21.6%)	
**Household Size**				**0.64**
** 0 to 3**	262 (35.3%)	112 (38.4%)	410 (36.2%)	
** 4 to 6**	376 (50.6%)	141 (48.3%)	561 (49.6%)	
** 7 or more**	105 (14.1%)	39 (13.4%)	161 (14.2%)	

**Table 4:** Perceptions of surgery, stratified by patient experience.

	Bonanza	Rosita	Total	P-Value
Number of Respondents	743	292	1132	
**Health Post Visit**				0.019*
** Yes**	469 (63.1%)	160 (54.8%)	669 (59.1%)	
** No**	274 (36.9%)	131 (44.9%)	462 (40.8%)	
** Missing**	0 (0%)	1 (0.3%)	1 (0.1%)	
**Hospital Visit**				**0.760**
** Yes**	595 (80.1%)	237 (81.2%)	917 (81%)	
** No**	148 (19.9%)	55 (18.8%)	215 (19%)	
**Transportation Cost**				0.002*
** <60 cordoba**	285 (38.4%)	67 (22.9%)	418 (36.9%)	
** 60 Cordoba or more**	292 (39.3%)	118 (40.4%)	429 (37.9%)	
** Missing**	166 (22.3%)	107 (36.6%)	285 (25.2%)	
**Transportation Type**				<0.001*
** Private**	137 (18.4%)	85 (29.1%)	231 (20.4%)	
** Public**	371 (49.9%)	131 (44.9%)	547 (48.3%)	
** Other**	87 (11.7%)	21 (7.2%)	139 (12.3%)	
** Missing**	148 (19.9%)	55 (18.8%)	215 (19%)	
**Transportation Time**				**0.240**
** Less than 1 hr**	341 (45.9%)	147 (50.3%)	556 (49.1%)	
** 1 hr or more**	254 (34.2%)	90 (30.8%)	361 (31.9%)	
** Missing**	148 (19.9%)	55 (18.8%)	215 (19%)	
**Public Transportation Available**				<0.001*
** Yes**	465 (62.6%)	247 (84.6%)	771 (68.2%)	
** No**	277 (37.3%)	45 (15.4%)	359 (31.7%)	
** Missing**	1 (0.1%)	0 (0%)	1 (0.1%)	
**Travel Obstacles Exist**				<0.001*
** Yes**	270 (36.3%)	58 (19.9%)	400 (35.3%)	
** No**	362 (48.7%)	205 (70.2%)	583 (51.5%)	
** Missing**	111 (14.9%)	29 (9.9%)	149 (13.2%)	
**Cellphone Use**				0.043*
** Yes**	519 (69.9%)	223 (76.4%)	802 (70.8%)	
** No**	224 (30.1%)	69 (23.6%)	330 (29.2%)	

Table 5 assesses key health related variables by perception of surgery both unadjusted and adjusted for key demographic characteristics. Individuals in each municipality who reported previously presenting to a health post were 1.4 times as likely to report a positive perception of surgery compared to those who had not visited a health post previously (p = 0.02). This association was consistent after adjustment for education, age, gender, and municipality. Although visiting a health post was associated with a positive perception of surgery, having previously visited a hospital had no significant effect on perception of surgery (OR = 0.93; p = 0.69) in unadjusted or adjusted models. Participants with higher travel costs were 28% less likely to report a positive perception of surgery compared to those with lower costs in the unadjusted model as well as adjusted for gender, education, rural/ urban, and age, however not after adjusting for site. Multiple travel related factors had counterintuitive relationships with perception of surgery. Participants with access to public transportation were 70% less likely to have a positive opinion of surgery compared to those who didn’t (OR = 0.31, p < 0.001). This result remained in all adjusted models, but moved towards the null when adjusted for municipality (OR = 0.52, p = 0.002). Those who reported having travel obstacles were 2.64 times as likely to have a positive opinion of surgery (p < 0.001). This result remained after adjustment for gender, education, rural/ urban, and age. However, it became insignificant after adjustment for site (OR = 0.89, p = 0.6) and was driven by the responses in Rosita. Longer travel time also showed an increased likelihood of a positive perception of surgery, however these results did not reach statistical significance. Cell phone usage went in the opposite direction expected, with individuals reporting having a cell phone being 30% less likely to have a positive opinion of surgery compared to those who did not (OR = 0.72, p = 0.04). This result was not significant after adjustment for education and site.

**Table 5:** Key health-related variables by perception of surgery.

*Variable*	Unadjusted		Adjusted for:							
			Education		Rural/Urban		Age		Municipality	
	OR (95% CI)	P-Value	OR (95% CI)	P-Value	OR (95% CI)	P-Value	OR (95% CI)	P-Value	OR (95% CI)	P-Value
**Health Post**	1.4(1.06–1.84)	0.016	1.38(1.05–1.82)	0.021	1.12 (0.81–1.55)	0.490	1.39(1.06–1.84)	0.017	1.71(1.25–2.34)	<0.001
**Hospital Visit**	0.93 (0.66–1.31)	0.690	0.97 (0.69–1.38)	0.910	1.07 (0.74–1.52)	0.720	0.93 (0.65–1.3)	0.670	0.83 (0.58–1.2)	0.33
**Travel Cost**	0.58(0.41–0.82)	0.0019	0.58(0.41–0.82)	0.002	0.52(0.34–0.78)	0.0019	0.57(0.41–0.81)	0.0017	1.29 (0.86–1.93)	0.21
**Public Transport**	0.31(0.22–0.44)	<0.001	0.31(0.22–0.44)	<0.001	0.23(0.16–0.34)	<0.001	0.31(0.21–0.44)	<0.001	0.52(0.35–0.79)	0.002
**Travel Obstacles**	2.64(1.9–3.7)	<0.001	2.59(1.87–3.63)	<0.001	2.67(1.92–3.75)	<0.001	2.64(1.9–3.7)	<0.001	0.89 (0.58–1.36)	0.6
**Travel Time**	1.22 (0.89–1.65)	0.21	1.13 (0.82–1.56)	0.44	0.84 (0.57–1.26)	0.42	1.22 (0.9–1.66)	0.21	1.37 (0.95–1.98)	0.09
**Cellphone Use**	0.72(0.52–0.98)	0.04	0.74 (0.54–1)	0.055	0.72(0.53–0.99)	0.043	0.71(0.51–0.96)	0.033	0.83 (0.59–1.15)	0.27

## Discussion

According to the Oxford English Dictionary, perception is defined as *the ability to become aware of something through the senses*, *or a way of regarding*, *understanding*, *or interpreting something; a mental impression* [12]. It follows that individuals reporting a positive or negative “interpretation” or perception of surgery are influenced by their level of exposure to the local healthcare system, in this case the availability of surgical services. Equally as important as an individual’s direct interaction with surgery, visualization of a friend’s or family member’s surgical experience as well as what an individual has heard through word of mouth from their community both have the potential to affect their perception of surgery. Conversely, visiting a hospital, having a cell phone, and difficulty reaching the hospital were counterintuitively associated with having a positive perception of surgery. This study highlights key relationships between patients within the Las Minas mining triangle of Nicaragua and the various factors that may affect their perception of surgery. Large scale data collection, capturing structural, financial, and cultural data should be used to help elucidate focused investments in areas most likely to improve utilization of healthcare and surgical services for patients in low resource settings.

The results of this study demonstrate that general healthcare exposure and barriers related to accessing surgical healthcare may be key factors in shaping an individuals’ perceptions of surgical care. Specifically, the finding that individuals who had previously visited a health post were 1.4 times as likely to report a positive perception of surgery compared to those who had not visited a health post previously (p = 0.016). This finding is consistent with previous literature highlighting the importance of how primary health care, including health posts, can improve health outcomes and promote positive perceptions of healthcare [13–15]. In addition to improving patients’ perception of care, primary healthcare can work to promote trust and satisfaction with the healthcare system [16]. In the context of rural Nicaragua, patients must have both access to surgical care and the willingness to do so. Even when appropriate investments in surgical services shore up supply, if patients do not subsequently seek care a supply-demand mismatch still exists. Therefore, a patient’s perception of surgery has implications on healthcare delivery, patient education, and a patient’s desire to seek specialized healthcare. Our data suggests that primary healthcare, by way of lower-level facilities such as health posts, may be a significant factor in helping improve a patient’s perception of surgery. If patient demand does not equal supply even in the presence of robust supply-side investment, there is an argument to be made that patient access has not truly been achieved.

The lack of significant difference in perception of surgery among individuals who had previously visited a hospital is likely more complex and multifactorial. Individuals seeking care within a hospital, rather than a health post, may perceive their condition to be more complex, need specialized or advanced care, and/or have urgent health care needs. When there is an acute change in health status, individuals will usually visit higher levels of health care [17]. A study by El-Haddad et al. describes how specific expectations of patients seeking care within a hospital are affected by their individual health outcomes, interaction with clinicians, and the patient’s association of the hospital with the health system’s overall capabilities [5]. These components of care delivery within a hospital, coupled with an expectation that the care they receive will adequately address their health needs may create an unfair expectation of care delivery compared to other healthcare environments, such as health posts. Dawkins et al. reported that while in high-income countries there is a greater emphasis on patient satisfaction, patient experience in low-middle-income countries is more closely related to the physical presence or absence of care [18]; like what is available within the limited capacity setting of the 3 district level hospitals within our study area, Las Minas, Nicaragua. These insights may help account for the varied responses that individuals who had visited a hospital provided when asked about their overall perception of surgery within our study. Further investigation could help elucidate specific patient populations, as related to health status, and their perception of surgical care within specific health care environments.

The finding that individuals with reported travel obstacles had a more positive perception of surgery compared to those utilizing public transportation, paying more than a normal bus fare for healthcare visits, or having access to a cell phone is surprising and somewhat counterintuitive. It is reasonable to expect frustrations with utilization of the public transport system for accessing healthcare services. Varela et al. reported that individuals using public transport for accessing healthcare reported having significantly longer distances to travel and needing to use multiple forms of transportation to reach their health destination [19]. Buzza et al. describes the burden that individuals experience when travelling for health care services is relative and directly related to the care they are receiving [20]. Long distances for routine care, such as lab work or wound care, are burdensome as compared to individuals with the viewpoint that travelling long distances for specialty care, such as cardiology or neurology, is necessary and expected. Both the World Disability Report and the World Health Survey have indicated that costs related to visiting health care are the most relevant barrier and frequent barrier, respectively [21,22]. Financial, transportation infrastructure, and geographical barriers all play a role in an individual’s ability to access healthcare. These factors, when non-congruent with an individual’s expectations, can negatively impact accessibility and allow for an intuitive connection to why transportation costs, above that of a normal bus fare, could be directly associated with a negative perception of surgery.

Cell phones potentially circumvent long distances and extended waiting times related to accessing healthcare services. Cell phones can also serve to increase accessibility to information, education and communication enabling the ability to better navigate barriers to surgical access [23]. However, despite cellphone access typically having a positive impact on individuals’ ability to interact with healthcare systems, our findings may represent a symptom of health misinformation that exists online. Within Latin America and Caribbean, 97% of the population is covered by mobile broadband, with 62% of the population using their connectivity to access the internet [24]. As mobile internet access continues to rise, so does health information seeking behavior on mobile devices [25]. Despite increased access to health information, available resources have been found to have high variability in quality [26]. Such findings may suggest a public health need for improved community eHealth literacy, increased internet engagement by physicians as well as creation and dissemination of accurate information. Further research to identify the underlying cause of this finding and potential means of resolution is recommended.

Travel obstacles were shown to have a strong association with a positive perception of surgery. A potential explanation of the finding is that individuals reporting travel obstacles have a higher appreciation for specialized care, such as surgery, due to the difficulty in accessing it if needed. Alternatively, those with travel obstacles could have more severe health conditions due to obstacles causing an inability to consistently access health care and giving these individuals a greater appreciation for needing surgical care. Interestingly, having a travel obstacle remained significant upon adjustment for factors such as education, location (rural/urban), and age. However, when assessing the influence of the municipality, the data indicates that the null hypothesis should be accepted as no significant association was found. Upon further stratification, this result was primarily driven by participant responses in Rosita, as there was a strong negative perception of surgery (47.4%), compared to Bonanza (9.1%) and Siuna (25.8%). Interestingly, the district hospital in Rosita has the lowest capacity to provide surgical care of the 3 hospitals in the region. Comparatively, the hospital in Rosita has the least hospital beds, including total surgical beds, no ICU level care, only 1 operating room (compared to 2 each in Siuna and Bonanza), and the least number of surgeons, anesthesiologists and obstetricians across the 3 hospitals. As previously discussed by Dawkins et al. [18], it is not difficult to infer that with the limited capacity of the hospital in the municipality of Rosita, if patients present with expectations of their health care needs being addressed, especially related to surgery, but are turned away or not provided care in a timely manner, this physical absence of care could directly impact their perception of surgery in a negative manner.

This study had several limitations. The first limitation lies in combining neutral and negative responses together regarding perceptions of surgery. The authors were interested in assessing true positive perception compared to all other responses. Neutral responses may be utilized when respondents don’t have enough context regarding the question, are not motivated to respond to the question, or worry about not verbalizing a socially acceptable response, especially when being asked by a well known surgical organization, such as Operation Smile. The largest limitation is the limited nature of the survey questions without the ability to further investigate or determine the causality of the discovered associations, as would potentially be possible through connecting a participants health status with their responses. With this limitation in mind, only 2 variables, previously visiting a health post and having to use public transportation to access health care, remained significant in the same direction upon adjustment across all 3 municipalities, thus signifying the strength of the association between these specific variables and their impact on an individual’s perception of surgery. Additionally, there could have been potential biases in self-reporting or non-standardization in survey questioning. Our study group attempted to preemptively account for these aforementioned limitations through the large number of participants surveyed and the pre-survey preparation of each survey/data collector, in addition to the multiple rounds of translation between local dialects, Spanish and English to assure consistency across survey questions. Furthermore, our study had a very focused geographical scope, and it is not known whether these findings would be generalizable to other populations, both within Nicaragua or globally, without further investigation. Despite these limitations, our data did highlight several intriguing insights and suggest the need to further investigate the underlying factors causing both significant and non-significant associations between healthcare environments, barriers to accessing care and the perception of surgery.

## Conclusion

In conclusion, our study attempts to investigate how different health environments, pre-hospital barriers, and access to technology impact an individual’s perception of surgery and may also affect patient demand for surgical services. Our findings suggest that individuals in the studied region who previously interacted with only lower-level healthcare environments, such as health posts, were significantly more likely to have a positive perception of surgery, compared to individuals who previously visited hospitals. Additional unexpected or counterintuitive findings showcased that access to public transport, transportation cost greater than $2USD, and cell phone usage increased the likelihood of having a negative perception of surgery. These findings provide insight into the complexity of how perceptions of healthcare services are impacted by both prehospital barriers to care and access to healthcare information. Further in-depth analysis of the local environment and stakeholders may help improve the success of cost-effective, targeted investments to yield high-impact results, especially in remote and underserved settings where funding is limited.

## Supporting information

S1 Checklist STROBE statement—Checklist of items that should be included in reports of *cross-sectional studies*. (DOCX)
